# Genotype Diversity of H9N2 Viruses Isolated from Wild Birds and Chickens in Hunan Province, China

**DOI:** 10.1371/journal.pone.0101287

**Published:** 2014-06-30

**Authors:** Ba Wang, Zhihua Liu, Quanjiao Chen, Zhimin Gao, Fang Fang, Haiyan Chang, Jianjun Chen, Bing Xu, Ze Chen

**Affiliations:** 1 State Key Laboratory of Virology, Center for Emerging Infectious Diseases, Wuhan Institute of Virology, Chinese Academy of Sciences, Wuhan, Hubei, China; 2 College of Life Sciences, Hunan Normal University, Changsha, Hunan, China; 3 Shanghai Institute of Biological Products, Shanghai, China; 4 Department of Environmental Science and Engineering, Tsinghua University, Beijing, China; Duke-NUS Gradute Medical School, Singapore

## Abstract

Three H9N2 avian influenza viruses were isolated from the Dongting Lake wetland, among which one was from fresh egret feces, the other two were from chicken cloacal swabs in poultry markets. Phylogenetic analyses suggested that eight genes of the egret-derived H9N2 virus might come from Korean-like or American-like lineages. The two poultry-derived H9N2 viruses were reassortants between the CK/BJ/94-like and G1-like viruses. Except the PB1 genes (90.6%), the nucleotide sequence of other internal genes of the two viruses exhibited high homology (>95%). In addition, they also exhibited high homology (96–98.3%) with some genes of the H7N9 virus that caused an epidemic in China in 2013. Nucleotide sequence of the poultry-derived and egret-derived H9N2 viruses shared low homology. Infection studies showed that the egret-derived H9N2 virus was non-pathogenic to both mice and chickens, and the virus was unable to infect chickens even through 8 passages continuously in the lung. On the other hand, the chickens infected by poultry-derived viruses showed obvious clinical symptoms and even died; the infected mice showed no noticeable clinical symptoms and weight loss, but viruses could be detected in their lungs. In conclusion, for the egret-derived H9N2 virus, it would take a long adaptation process to achieve cross-species transmission in poultry and mammals. H9N2 viruses isolated at different times from the same host species in the same geographical region presented different evolutionary status, and virus isolated from different hosts in the same geographical region exhibited genetic diversity. Therefore, it is important to continue the H9N2 virus surveillance for understanding their evolutionary trends so as to provide guidance for disease control and prevention.

## Introduction

The H9N2 avian influenza virus (AIV) was first isolated from North American waterfowls in 1966 and it has disseminated worldwide since then [1]. Many studies have shown the presence of the H9N2 viruses in both waterfowls and poultry and two-way transmission between them [2], [3]. Guan et al. found that the internal genes of the H5N1 AIV that caused human infections in Hongkong in 1997 originated from H9N2 subtype AIV [4]. The first human case infected by H9N2 AIV occurred in 1999 [5]. Studies have demonstrated that the characteristics of the H9N2 virus might change after genetic reassortment through multiple hosts. Therefore, it is important to strengthen the monitoring and tracking studies of H9N2 AIV.

Epidemiology and phylogenetic studies found that the viruses that caused H9N2 AIV outbreak in Hong Kong live poultry market could be divided into three distinct lineages: A/Chicken/Beijing/1/94 (Ck/BJ/94-like), Quail/Hong Kong/G1/97 (G1-like) and Duck/Hong Kong/Y439/97 (Y439-like or Korean-like) [6], [7]. In mainland China, H9N2 AIV was first isolated from chickens in Guangdong province in 1994 [8]. The Ck/BJ/94-like and G1-like lineages have been prevalent in domestic poultry, such as quails and chicken, in southern China since the early 1990s [4], [7], [9]. The Y439-like lineage was isolated from ducks in Hong Kong in 1997 and was also isolated from sick chickens in South Korea in 1996. In mainland China, monitoring data in recent years indicated that novel genotypes of H9N2 AIV had kept emerging [10], [24]. More importantly, six gene segments of H7N9 AIV that caused human infections in 2013 came from H9N2 viruses, which again demonstrated that the H9N2 AIV was a potential threat to humans [11].

A total of three H9N2 subtype AIV strains were isolated during 2010–2012. Of which one was isolated from fresh egret feces collected in Dongting Lake Egret Nature Reserve, the other two were isolated from chicken cloacal swabs collected in poultry markets. We carried out whole-genome sequencing for the three viruses, constructed phylogenetic trees and investigated the sources of the virus genomes. Pathogenicity tests of these H9N2 viruses were conducted in mice and SPF white Leghorn chickens. The study revealed the evolutionary status and likelihood of cross-species transmission of H9N2 AIVs in wide birds and poultry in Dongting Lake wetland, and contributed data for molecular epidemiology of AIV.

## Materials and Methods

### Ethics Statement

All animal experiments were approved by the Institutional Animal Care and Use Committee (IACUC) of Laboratory Animal, Wuhan Institute of Virology, Chinese Academy of Sciences (Approval numbers: WIVA04201202 for mice and WIVA05201001 for chickens). This study was carried out in accordance with the recommendations in the Guide for the Care and Use of Laboratory Animals. All procedures were performed under anesthesia. Animals were closely monitored and observed for development of disease once daily, and all efforts were made to minimize suffering.

Fresh migratory bird feces were collected at Dongting Lake Egret Nature Reserve (longitude: 112.819496, latitude: 29.514841) and no specific permissions were required for these locations. The cloacal swabs collected in poultry markets (longitude: 112.996925, latitude: 29.441994) and no specific permissions were required for these activities. All field studies did not involve endangered or protected species.

### Sample collection

Sterile cotton swabs were used to collect fresh migratory bird feces and poultry cloacal samples. The swabs were placed into viral transport media. All the samples were placed in a hand-held portable 4°C refrigerator, transported to the laboratory within 24 hours, and frozen in −80°C immediately for future use.

### Virus isolation

The swabs frozen in the −80°C freezer were transferred to a 4°C refrigerator for thawing. Then the sample was spun at 6000 rpm for 10 min at 4°C, and the supernatant was inoculated into 10-day-old SPF embryonated chicken eggs. The embryonated chicken eggs were cultured at 37°C for 48–72 hours. The embryos that died within the first 24 hours were discarded, for the rest live or dead embryos, allantoic fluid was collected and spun at 3000 rpm for 10 min at 4°C and then tested for hemagglutination. The allantoic fluid positive for hemagglutination was divided into aliquots and frozen at −80°C for further tests.

### RNA extraction and nucleotide sequencing

RNA extraction and reverse transcription of virus RNA were performed according to the instructions of the RNA extraction kit (Takara) and mouse reverse transcription kit (MLV, Promega), respectively. Influenza virus gene fragments were amplified by PCR using a Phusion High-Fidelity PCR Kit (New England Biolabs). The primers used to amplify the eight genes were universal primers designed by Hoffman et al. [12]. The recovered gene fragments were cloned into pGEM-T vector and sequenced by the dideoxy method with an ABI 3730 DNA sequencer (Applied Biosystems). The complete genome sequences of influenza virus were edited and aligned with BioEdit version 7.0.5.2.

### Phylogenetic analysis

Phylogenetic analysis was based on nucleotides 94–1,556 (1,463 bp) of the HA gene, 38–1,371 (1,334 bp) of NA, 56–2,289 (2,234 bp) of PB2, 40–2,233 (2,194 bp) of PB1, 25–2,166 (2,142 bp) of PA, 39–1,388 (1,350 bp) of NP, 95–931 (837 bp) of M, and 38–806 (769 bp) of NS. Multiple alignments were constructed by using BioEdit (version 7.0.5.2). Phylogenetic trees were generated by using Maximum Likelihood analysis with 1000 bootstrap replicates in the MEGA program (version 5.2).

### Chicken study

Two routes of virus infection, intravenous (i.v) and intranasal (i.n) injection were used. For each route, 6-week-old SPF white Leghorn chickens were divided into two groups, eight each group. In i.v groups, virus was diluted 10 fold and then injected intravenously to the chickens at a dose of 0.2 ml each. In i.n groups, each chicken was anesthetized via intramuscular injection of ketamine (20 mg/kg) and xylazine (1 mg/kg) and given a dose of 10^6^EID_50_ virus in 0.1 ml. Oropharyngeal and cloacal swabs were collected on day 1, 3, 5, 7 p.i.. Viral titration was conducted in 10-day-old SPF embryonated chicken eggs. The chickens were observed for 21 days post-inoculation (p.i) and their symptoms and survival were recorded. The symptoms, virus titration of lungs and death were as results of virus infection.

For lung passage experiment in SPF white Leghorn chickens, eight chickens were infected intranasally with each virus in a volume of 0.1 ml of 10^6^EID_50_ virus after being anaesthetized. Three days after infection, the chickens were euthanized with ketamine and xylazine, and the lungs were taken out and homogenized with a total of 2 ml of PBS containing 0.1% BSA. The tissue homogenate was used for virus titration after removing cellular debris by centrifugation. Virus titration was performed as above. The remaining five chickens were observed for symptoms and survival for 21 days and post-recovery sera were collected for hemagglutination inhibition (HI) test. The same procedure was performed from Passage 0 (P0) to 8 (P8).

### Mouse study

Specific-pathogen-free female BALB/c mice, aged 6 to 8 weeks old, were purchased from the Center for Disease Control and Prevention in Hubei Province, China. Mice were bred in the Animal Resource Center at the Wuhan Institute of Virology, Chinese Academy of Sciences. All mice were maintained in specific-pathogen-free conditions. The BALB/c mice were divided into three groups, including two experimental groups and one control group, twenty-one each group. For the experimental groups, each mouse was anaesthetized with pentobarbital sodium and infected intranasally with 10^6^ EID_50_ virus in 50 µl. The mice (n = 12) were observed for 21 days p.i. and their symptoms, mortality and weight loss were recorded. On day 3, 5 and 7 p.i., three mice per group were euthanized, and the lungs, brains, spleens and kidneys were taken out and homogenized with a total of 2 ml of PBS containing 0.1% BSA.

### Histopathological analysis

For histopathological analysis, lungs of SPF chicken were removed immediately following euthanasia, and fixed with 10% formalin at 4°C. Subsequently, lungs were stained with Hematoxylin and Eosin (H&E), and examined for pathological changes corresponding to infection. Images were obtained on an Nikon80i light microscope at 400-fold original magnification.

### Antigenic analysis

Sera were harvested from the four groups of inoculated chickens on day 21 p.i. for confirmation of seroconversion and antigenic analysis by HI assays with 1.0% chicken erythrocytes according to relevant recommendation of OIE.

### Nucleotide sequence accession numbers

The nucleotide sequences obtained in this study are available from GenBank under accession numbers JX437684 to JX437691, KF714772- KF714787.

## Statistics

The results of the test groups were evaluated by Student's t-test; if p-value is less than 0.05, the difference was considered significant. The survival rates of the mice in the test and control groups were compared by using Fisher's exact test.

## Results

### Virus isolation and homology analysis

From October 2010 to October 2012, three H9N2 subtype AIVs were isolated from Dongting Lake wetland. One virus was isolated from fresh egret fececs collected in Dongting Lake Egret Nature Reserve and named A/Egret/Hunan/1/2012 (H9N2). The other two viruses were isolated from cloacal swabs collected in poultry markets and named A/Chicken/Hunan/12/2011(H9N2) and A/Chicken/Hunan/1/2012 (H9N2), respectively.

The whole genomes of the three viruses isolated in this study were sequenced and then nucleic acid and protein sequence homology were compared (Table 1). Genes of the egret-derived H9N2 virus (A/Egret/Hunan/1/2012) exhibited high homology with the corresponding genes of different subtypes viruses. Its HA gene had 97% nucleic acid homology with that of A/northernshoveler/Interior Alaska/8BM3470/2008 (H9N2), a North American wild bird strain. NA gene showed homology of 98% with that of the Snow goose/Montana/466771-4/2006(H5N2) strain. The PB1 and PB2 genes showed high homology (99%) with those of WD/Korea/SNU50-5/09(H5N1) strain. PA gene had 99% nucleic acid homology with that of Nor-sv/California/2810/11(H11N2) strain and NS gene had 99% homology with that of Surface water/Minnesota/W07-2241/2007(H3N8) strain. M gene showed 99% nucleic acid homology with that of A/Duck/Hunan/S4013/2011(H11N9) and A/wild duck/Korea/CSM4-28/2010(H4N6) strains respectively. NP gene had 98% homology with that of WD/Korea/SH5-60/08(H4N6) strain.

**Table 1:** Comparisons of three H9N2 viruses with isolates in GenBank of highest nucleotide and amino acid identity (%).

Gene	Virus*	Site	Homologous virus^a^	Homology (%)	Site	Homologous virus^b^	Homology(%)
	E1	Jan-41	WD/Korea/SNU50-5/09(H5N1)	98	1-768	CK/Netherlands/1/03(H7N7)	99
PB2	C1	Jan-41	CK/China/AH-10-01/10(H9N2)	98	1-768	CK/Netherlands/1/03(H7N7)	99
	C12	Jan-80	CK/HB/1102/10(H5N2)	95	1-759	CK/HB/1102/2010(H5N2)	99
	E1	Jan-41	WD/Korea/SNU50-5/09(H5N1)	98	1-757	DK/Hokkaido/Vac-2/04(H7N7)	99
PB1	C1	Jan-41	WD/Korea/SH5-26/08(H4N6)	98	1-757	WG/Mongolia/1-125/08(H3N8) CK/Jiangsu/Q3/10(H9N2)	99
	C12	Jan-74	CK/HB/1102/10(H5N2)	99	1-757		99
	E1	Jan-08	Nor-sv/California/2810/11(H11N2)	99	1-716	MD/Alberta/569/08(H1N1]	99
PA	C1	Jan-33	CK/China/AH-10-01/10(H9N2)	99	1-716	SW/Taizhou/5/08(H9N2)	99
	C12	Jan-62	EQ/GX/3/11(H9N2)	98	1-716	CK/Zhejiang/607/11(H9N2)	99
	E1	1-1717	Nor-sv/IA/8BM/08(H9N2)	97	1-560	Nor-sv/IA/8BM/08(H9N2)	97
HA	C1	1-1742	CK/SD/C/09(H9N2)	98	1-560	CK/Anhui/B11/10(H9N2)	99
	C12	1-1683	CK/Anhui/WBQ/11(H9N2)	99	1-560	CK/Anhui/WBQ/11(H9N2)	99
	E1	1-1565	WD/Korea/SH5-60/08(H4N6)	98	1-504	SW/Fujian/1/03(H5N1)	99
NP	C1	1-1594	CK/China/AH-10-01/10(H9N2)	99	1-498	CK/HB/4/08(H9N2)	99
	C12	1-1594	CK/China/AH-10-01/10(H9N2)	99	1-498	CK/HB/4/08(H9N2)	99
	E1	1-1441	SG/Montana/466771-4/06(H5N2)	98	1-469	SG/MTN/466771-4/06(H5N2)	99
NA	C1	1-1432	CK/China/AH-10-01/10(H9N2)	98	1-466	CK/Tongshan/1/11(H9N2)	99
	C12	1-1432	CK/China/AH-10-01/10(H9N2)	98	1-466	CK/Shandong/B2/07(H9N2)	97
	E1	1-1027	WD/Korea/CSM4-28/10(H4N6)	99	1-252	WD/Korea/CSM4-28/10(H4N6)	99
M	C1	1-1043	CK/China/AH-10-01/10(H9N2)	99	1-252	CK/China/AH-10-01/10(H9N2)	99
	C12	1-1043	CK/China/AH-10-01/10(H9N2)	99	1-252	CK/China/AH-10-01/20(H9N2)	99
	E1	1-889	SF/MST/W07-2241/07(H3N8)	99	1-217	SF/MST/W07-2241/07(H3N8)	99
NS	C1	1-883	CK/SD/03/10(H9N2)	99	1-217	CK/SD/03/10(H9N2)	99
	C12	1-919	CK/China/AH-10-01/10(H9N2)	98	1-217	CK/China/AH-10-01/10(H9N2)	97

Nor-sv, northern shoveler; EQ equine; SG snow goose; SF, surfacewater; SW, swine; MD, mallard; WG, whitegoose; HB, Hubei; GX, Guangxi; SD, Shandong;

MST, Minnesota; MTN, Montana.

*E1: A/Egret/Hunan/1/2012. C1: A/Chicken/Hunan/1/2012. C12: A/Chicken/Hunan/12/2011.

a Nucleotide sequence isolate with the highest homology.

b Amino acid sequence isolate with the highest homology.

The HA and NA genes of the A/Egret/Hunan/1/2012 virus had about 83% homology with those of A/Chicken/Hunan/12/2011 and A/Chicken/Hunan/1/2012 viruses and homology of the NS gene was even lower (70%). The genes of two poultry-derived viruses shared high homology except the PB1 gene. Nucleotide homology of the PB1 gene between the two poultry viruses was only 90.6%, lower than the PB1 gene homology between A/Chicken/Hunan/1/2012 and A/Egret/Hunan/1/2012 (96.8%). Some genes of the two poultry viruses also had high homology with those of the H7N9 virus isolated from pigeons in Shanghai in 2013 (e.g., M gene: 98.3%) (Table 2).

**Table 2:** Homology (%) of nucleotide sequences of eight genes of virus from chickens and relevant sequences of birds or H7N9.

Virus^a^	Percent identity of nucleotide (%)																							
	HA			NA			PB2			PB1			PA			NP			M			NS		
	E1	BJ16	S69	E1	BJ16	S69	E1	BJ16	S69	E1	BJ16	S69	E1	BJ16	S69	E1	BJ16	S69	E1	BJ16	S69	E1	BJ16	S69
C1	83.8	97.4	-	84.5	95.2	-	87.8	96.0	95.5	96.8	90.0	90.0	90.2	96.5	96.4	90.7	95.8	95.8	90.6	99.0	98.3	69.7	95.2	95.8
C12	83.7	97.4	-	84.6	95.2	-	86.2	96.9	96.5	90.3	97.1	98.5	89.9	96.8	96.6	90.9	96.1	95.9	90.4	98.7	98.0	70.7	99.0	97.6

a E1: A/Egret/Hunan/1/2012; C1: A/Chicken/Hunan/1/2012; C12: A/Chicken/Hunan/12/2011;

BJ16:A/Brambling/Beijing/16/2012(H9N2); S69: A/Pigeon/Shanghai/S1069/2013(H7N9).

The identity was not done.

### Phylogenetic analysis

**Figure 1:**
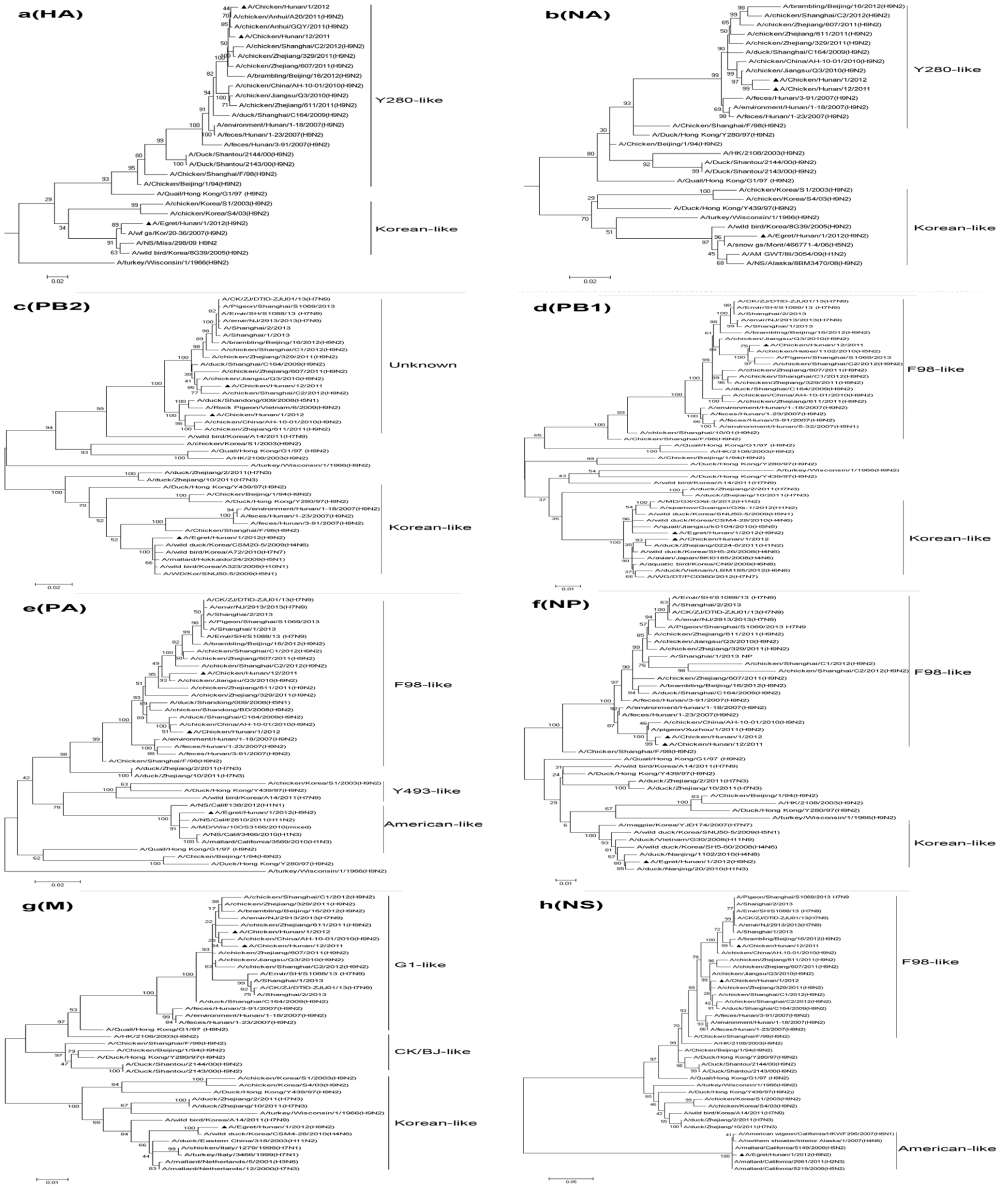
Phylogenetic trees for the HA, NA, PB2, PB1, PA, M, NS, NP genes of the H9N2 influenza A virus isolated in present study. Trees were generated by using Maximum Likelihood analysis with 1000 bootstrap replicates in the MEGA program (version 5.2).

Phylogenetic analysis indicated that the HA and NA genes of the two poultry-derived viruses belonged to the CK/Bei/94-like lineage, which had two subgroups represented by A/Duck/HongKong/Y280/97 and A/Chicken/Shanghai/F/98, respectively. The HA genes of the two poultry-derived viruses belonged to the subgroup represented by A/Duck/HongKong/Y280/97 (Figure1a, b). Except PB1 and PB2 genes, all the other gene segments were in either the CK/Bei/94 lineage or the G1 lineage (Figure1c-h, Table 3). The PB2 genes of these two H9N2 viruses shared high homology but they were in the DK1-like lineage. The PB1 genes of the two chicken H9N2 viruses were in different evolutional lineages; A/Chicken/Hunan/12/2011 virus belonged to the CK/Bei/94-like lineage, while A/Chicken/Hunan/1/2012 virus belonged to the Korean-like lineage. For A/Egret/Hunan/1/2012 virus, its NS and PA genes belonged to the American-like lineage and the other genes originated from the Korean-like influenza viruses (Table 3).

**Table 3:** Gene constellation of H9N2 viruses isolated in this study.

Virus^a^	Lineage of gene segments^b^							
	HA	NA	PB2	PB1	PA	NP	M	NS
C1	Ck/Bei	Ck/Bei	Dk1	Korean-like	Ck/Bei	Ck/Bei	G1-like	Ck/Bei
C12	Ck/Bei	Ck/Bei	Dk1	Ck/Bei	Ck/Bei	Ck/Bei	G1-like	Ck/Bei
E1	Korean-like	Korean-like	Korean-like	Korean-like	American-like	Korean-like	Korean-like	American-like

a E1: A/Egret/Hunan/1/2012 C1: A/Chicken/Hunan/1/2012 C12: A/Chicken/Hunan/12/2011.

b Ck/Bei: A/Chicken/Beijing/1/94; G1-like: Quail/Hong Kong/G1/97; Dk1: Dk/ST/7488/04.

### Molecular characterization

Amino acid sequence analysis revealed the connecting peptide of HA was ASDR/G in A/Egret/Hunan/1/2012 virus and RSSR/G in the two chicken isolates (Table 4). The RSSR/G motif was consistent with the sequence of poultry H9N2 viruses prevalent in mainland China. In addition, each of the three sequences had one or two basic amino acids, which showed a feature of low pathogenic viruses [9], [13]. The three isolates shared the same amino acid sequences at their HA receptor-binding site, having leucine (L) at position 226 (H3 numbering) and glycine (G) at position 228, which indicated that their HA proteins favor binding with α-2,6-linked sialic acid receptors and the possibility of the three isolates to infect humans [14]. The analysis of surface glycoproteins potential glycosylation sites revealed that the HA protein of A/Egret/Hunan/1/2012 and A/Chicken/Hunan/1/2012 viruses had eight potential glycosylation sites (positions: 29, 82, 141, 218, 298, 305, 492, 551), which were the same as most H9N2 viruses [15], whereas the HA protein of A/Chicken/Hunan/12/2011 virus had an additional glycosylation site at position 331. NA protein of A/Egret/Hunan/1/2012 virus did not have the 3-amino acid deletion at positions 63-65 in the stem region while the NA proteins of the other two isolates had the 3-amino acid deletion at positions 63-65, which leaded the deletion of one potential glycosylation site. It has been reported that this amino acid deletion might be necessary for virus adaptation from wild birds to poultry (chicken) [16]. The NA proteins of the three viruses also differed in glycosylation sites. The NA protein of A/Egret/Hunan/1/2012 virus had seven potential glycosylation sites (positions: 61, 69, 86, 146, 200, 234, 402); A/Chicken/Hunan/12/2011 virus had six potential glycosylation sites (positions: 66, 83, 143, 197, 231, 261), and A/Chicken/Hunan/1/2012 virus had seven potential glycosylation sites (positions: 44, 66, 83, 143, 197, 231, 261). The transmembrane region of the M2 protein of A/Egret/Hunan/1/2012 virus did not have single amino acid mutations (e.g., 26L, 27V, 30A, 31S, 34G, 37H, and 41W), suggesting sensitivity of the strain to M2 ion channel inhibitors [17]. In contrast, the two chicken isolates both had the S31N amino acid substitution, suggesting that they might have gained resistance to M2 ion channel inhibitors. The D92E substitution of NS1 protein could enhance virus resistance, but it was not present in any of the three isolates [18].

**Table 4:** Molecular characterizations of HA, NA, PB2, PB1, PA, NP, M and NS at representative sites.

Virus^a^	HA		NA	PB2		PB1-F2	PA	M2								NS1
	Connecting peptide	RBS^a^	Stalk deletion (63-65)	627	701	66	356	372	26	27	30	31	34	37	41	92
**E1**	ASDR↓G	LSG	-	E	D	N	K	E	L	V	A	S	G	H	W	D
**C1**	RSSR↓G	LSG	+	E	D	N	K	E	L	V	A	N	G	H	W	D
**C12**	RSSR↓G	LSG	+	E	D	S	K	E	L	V	A	N	G	H	W	D

a E1: A/Egret/Hunan/1/2012; C1: A/Chicken/Hunan/1/2012; C12: A/Chicken/Hunan/12/2011.

-: There was no deletion. +: There was deletion.

For the polymerase complex, no amino acid substitution was found in sites related to host specificity and viral replication (e.g., E627K and D701N in PB2, L356R in PA), indicating that these three isolates were low pathogenic to mammal and avian hosts [18]-[21]. The PB1-F2 protein of A/Chicken/Hunan/12/2011 virus had the N66S substitution, which indicated this isolate might have enhanced virulence, while the mutation was not found in the other two isolates.

### Replication of H9N2 viruses in chickens

A/Egret/Hunan/1/2012 and A/Chicken/Hunan/12/2011 viruses were selected for animal experiments. Chickens were infected via two routes, intravenous (i.v) or intranasal (i.n) injection. The chickens infected with A/Chicken/Hunan/12/2011 virus via either route presented similar clinical symptoms. They started showing hoarse voices and listlessness from day 3 p.i.; one chicken in the intranasal group died on day 5 p.i., then they started to recover on day 9 p.i.. Virus shedding was observed for most infected chickens, and virus could be detected in both pharynges and cloaca. Viral load in pharynges and cloaca peaked on day 3 p.i. in both route groups. On day 7 p.i., virus test was negative in the cloaca for all the chickens, but pharyngeal samples from most chickens were still positive. Chickens inoculated with A/Egret/Hunan/1/2012 virus via either route did not present significant clinical symptoms and no chicken died. Virus was detected only in one chicken of the intranasal group in both pharyngeal and cloacal samples on day 1 p.i., all the other chickens were tested negative for virus at all the time points post-infection. The chicken number of virus shedding in A/Chicken/Hunan/12/2011 virus-infected was far more than that of A/Egret/Hunan/1/2012 virus- infected (*p*<0.05) (Table 5).

**Table 5:** Pathogenicity and replication of the H9N2 viruses in chickens.

Vrius^a^	Infection route	Days post- infection	Oropharyngeal		Cloacal		No.of survivors/no. tested
			No. of shedding virus/no.tested	Virus titer^b^	No.of shedding virus/no.tested	Virus titer^b^	
**C12**	Intravenous	1	6/8^c^	1.3±0.0^c^	3/8	1.0±0.6^c^	8/8
		3	8/8^c^	1.7±0.7^c^	5/8^c^	1.4±0.7^c^	
		5	8/8^c^	1.3±0.6^c^	1/8	1.0±0.0^c^	
		7	5/8^c^	1.0±0.0^c^	0	0	
	Intranasal	1	7/8^c^	1.4±0.7	3/8	1.0±0.0^c^	7/8
		3	7/8^c^	3.1±0.2^c^	8/8^c^	2.2±0.2^c^	
		5	7/8^c^	2.8±0.4^c^	6/8^c^	1.7±0.6^c^	
		7	5/7^c^	1.8±0.4^c^	0	0	
**E1**	Intravenous	1	0	0	0	0	8/8
		3	0	0	0	0	
		5	0	0	0	0	
		7	0	0	0	0	
	Intranasal	1	1/8	1.0±0	1/8	1.0±0	8/8
		3	0	0	0	0	
		5	0	0	0	0	
		7	0	0	0	0	

One group of eight six-week-old specific-pathogen-free white leghorn chickens were inoculated intravenously with 0.2 ml of 1∶10-diluted virus stock and observed for 14 days after infection.

a E1: A/Egret/Hunan/1/2012 C12: A/Chicken/Hunan/12/2011.

b Virus Titer: log_10_ EID_50_/ml, mean ±SD.

c Significant difference (*p*<0.05).

Chicken lung tissue pathology revealed obvious pulmonary inflammatory responses on day 3 and 7 p.i. in chickens infected with the poultry-derived H9N2 virus. Partial necrosis appeared in lung tissues on day 3 and 7 p.i., more severe on day 3. The results indicated that poultry-derived H9N2 virus could not only replicate effectively in chickens, but also cause cellular inflammatory response and lung tissue injury. In contrast, the egret-derived H9N2 virus was less pathogenic to chickens than chicken-derived virus (Figure S1). The histopathology findings were consistent with the results of lung virus titer.

In addition, A/Egret/Hunan/1/2012 virus was 8 passaged continuously in the lungs of SPF white Leghorn chickens. No chicken showed overt clinical symptoms (such as hoarse voice and listlessness) and they kept normal food and water intake. The P0 to P8 chicken lung homogenate were inoculated into SPF eggs and cultured for 48–72 hours in a 37°C incubator and the allantoic fluid of chicken embryos were performed hemagglutination test. The virus was detected only in the P0 sample, no virus were detected in lungs of egret-derived H9N2 virus-infected at 3d p.i. and no antibody was detected at 21d p.i. for the rest 8 passages.

All chickens infected with A/Chicken/Hunan/12/2011 virus had seroconversion on day 21 p.i with very high HI titer. In contrast, none of the chickens inoculated with A/Egret/Hunan/1/2012 virus, either via i.v or i.n, showed seroconversion (positive if HI>16).

### Replication of H9N2 viruses in mice

**Figure 2:**
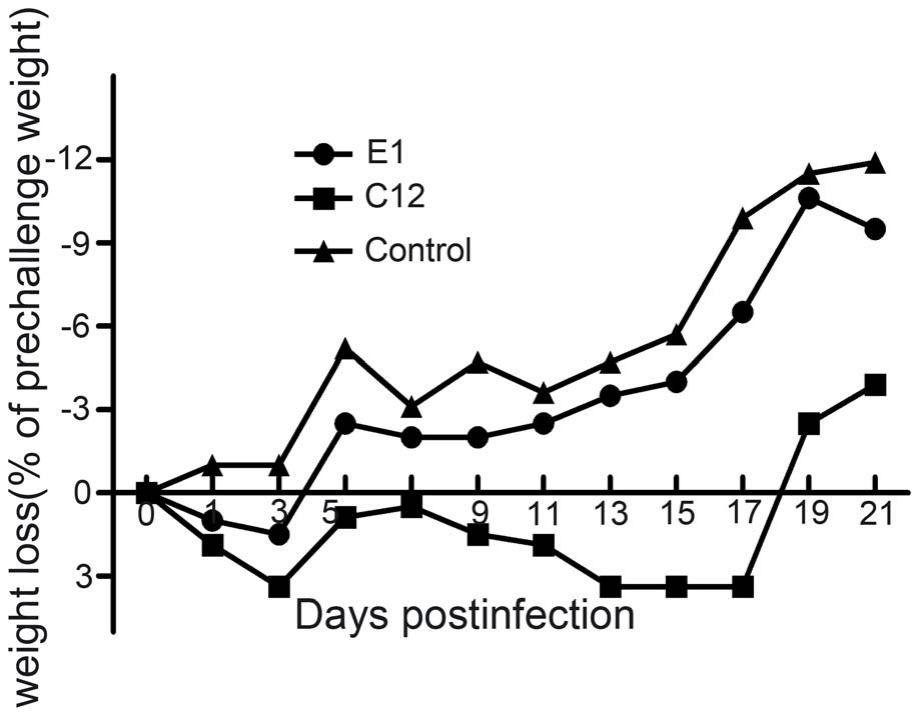
Bodyweight changes after challenge with the viruses. BALB/c mice were intranasally inoculated with A/Egret/Hunan/1/2012, A/Chicken/Hunan/12/2011 viruses at 1×10^6.5^ EID_50_. The bodyweights of 10 mice in each group were measured daily from the date of challenge to 21 days after challenge. Values represent mean ± SD of each group of mice.

A/Egret/Hunan/1/2012 and A/Chicken/Hunan/12/2011 viruses were also used for mice infection experiments. None of the mice showed overt symptoms and all mice survived. The egret-derived isolate group showed slight weight loss, whereas the poultry-derived isolate group showed symptoms such as slight piloerection and trembling and more weight loss as compared to the egret-derived isolate group. Their weight started to rebound from day 7 p.i. (Figure 2).

Virus detection was performed on lung, brain, spleen and kidney tissues taken on day 3, 5 and 7 p.i.. The results indicated only the poultry-derived virus could replicate in the lungs of mice, but the viral load was low and the result was positive only on day 3 and 5 p.i.. No virus was detected in the other organs. The egret-derived virus did not replicate in the lungs of mice and virus detection was negative for the other organs (Table 6).

**Table 6:** Replication of the H9N2 viruses in mice.

Virus	Days post- infection	Virus titer [log_10_(EID_50_/ml±SD)]			
		Brain	Lung	Spleen	Kidney
C12	3	-a	2.2±0.9^b^	-a	-a
	5	-a	2.8±0.4^b^	-a	-a
	7	-a	-a	-a	-a
E1	3	-a	-a	-a	-a
	5	-a	-a	-a	-a
	7	-a	-a	-a	-a

Six-week-old BALB/c mice were infected intranasally with 10^6.5^ EID_50_ of the viruses. Organs were collected on 3, 5, 7d after infection, and were titrated for virus infectivity in 10-day-old SPF embryonated chicken eggs.

E1: A/Egret/Hunan/1/2012 C12: A/Chicken/Hunan/12/2011.

a Virus was not detected in these samples.

b Significant difference (*p*<0.05).

### Antigenic analysis

Antisera were collected on day 21 post-infection. The cross-reactivity of the three viruses was investigated by HI assay. The antisera to A/Chicken/Hunan/12/2011 virus could cross-react well with A/Chicken/Hunan/1/2012 and A/Chicken/Hunan/12/2011 viruses (Table 7). The HI titer for antisera to A/Egret/Hunan/1/2012 virus reacting with the antigen virus was 16, suggesting no seroconversion following A/Egret/Hunan/1/2012 virus infection. Therefore, its antisera did not cross-react with the two chicken isolates. High HI titer was found when antisera were from chickens i.v infected with A/Chicken/Hunan/12/2011 virus reacting with A/Egret/Hunan/1/2012 virus.

**Table 7:** Antigenic analysis of H9N2 influenza viruses.

Virus	HI titers with post-infection antisera^a^			
	Intravenous		Intranasal	
	E1	C12	E1	C12
C12	-	5120	-	2560
C1	-	2560	-	1280
E1	16	20	-	40

E1: A/Egret/Hunan/1/2012 C1: A/Chicken/Hunan/1/2012 C12: A/Chicken/Hunan/12/2011.

The HI assay was started at a 1∶10 dilution.

a Antisera were first diluted tenfold. HI titers represent the reciprocal of the dilution that resulted in complete inhibition of agglutination of 1.0% chicken RBCs. HI>16 was positive.

-HI titers were not detected.

## Discussion

Last several years, human infections of H9N2 subtype avian influenza viruses have occurred, which have increasingly gained more attention [15], [22], [23]. Dongting Lake Wetland is a very important migratory habitat and wintering site for East Asian migratory birds, whereas poultry markets are hotbeds for AIVs and are closely related to human activities. In the present study, three H9N2 subtype AIVs were isolated. Phylogenetic tree of HA showed two chicken isolates clustered in the CK/BJ/94-like lineage, while the egret isolate belonged to Korean-like lineage.

In recent years, CK/BJ/94-like AIVs have been prevalent in poultry in central and southern China [10], [24]. The two chicken isolates of this study had high homology with H9 subtype viruses isolated from poultry in southern China and they were isolated from the same area at different times, showed different evolutionary status. Nucleotide sequence comparison showed some internal gene segments of the two H9N2 virus shared high homology with those of the H7N9 virus, and they also showed large differences among them. These results indicated that H9N2 AIVs from China's poultry markets presented complicated evolutionary characteristics.

Reports on H9N2 AIV isolated from wild birds have been rare recently. In the present study, one H9N2 virus was obtained from egrets in Dongting Lake, which, although clearly differed from the two poultry-derived H9N2 viruses, but shared 96.8% homology in PB1 nucleotide sequence with A/Chicken/Hunan/1/2012 virus, suggesting it exchanged gene segments with poultry-derived viruses.

Virus challenge experiments indicated that virus shedding occurred in most chickens infected with the poultry-derived viruses whereas the egret-derived virus could not replicate effectively in chickens. These findings indicated that the poultry-derived H9N2 viruses could not only adapt well in chickens, but also could shed virus into the environment, which increased the possibility of AIV transmission and reassortment among susceptible animals. In contrast, the egret-derived H9N2 virus was not yet capable of infecting chicken. The M gene plays a decisive role in cross-species transmission and pathogenicity of AIV. The M gene in the two poultry-derived H9N2 viruses belonged to the G1-like lineage, the same lineage as the M gene isolated from the Hongkong human H9N2 infection cases. Studies have shown that M gene is related to host range of influenza viruses and their adaptability to new hosts. Whether the M gene of the H9N2 poultry isolates in this study is directly related to cross-species transmission needs to be confirmed by further studies [25], [26].

All chickens infected with the chicken-derived isolates showed seroconversion and HI test results showed little antigenic variation between the two H9N2 viruses. No chickens infected with the egret-derived isolate showed seroconversion. Cross-reactivity was observed between antisera to the chicken-derived isolate and the egret-derived virus, although the titers were low, indicating the HA of A/Chicken/Hunan/12/2011 and A/Egret/Hunan/1/2012 viruses shared antigenic sites. Results of HI test and the HA gene phylogenetic analysis were consistent in that the two viral genomes had big differences.

Results from lung passage of the egret H9N2 virus in SPF chicken indicated that this virus could not cross-species transmit effectively. Chickens could clear the virus by their own immune system. The chicken-derived H9N2 viruses could replicate in the lungs of mice without adaptation (though the titers were low) whereas the egret-derived virus could not replicate in the lungs of mice. This finding indicated that it would take an ongoing adaptation process for un-adapted poultry H9N2 viruses to replicate stably and become pathogenic to mammalian hosts, and it would take even longer evolution for a wild bird derived H9N2 virus to adapt to mammals.

This study performed preliminary analysis of the biological characteristics of the H9N2 viruses isolated from different sources and found big differences between them, and it seemed that some more mutations and further evolution would be required for the wild-bird derived virus to adapt to poultry. These findings laid a foundation for further studies on virulence enhancement and molecular mechanism of pathogenesis of influenza viruses. The ongoing molecular epidemiology surveillance for H9 viruses in poultry-markets, lakes and wetlands is necessary for understanding their roles in the evolution of highly pathogenic influenza viruses and to prevent their potential pandemic in poultry and humans.

## Supporting Information

Figure S1 **Histopathological changes of lungs are shown by Hematoxylin and Eosin (H&E) staining after virus infection.** A, infected by E1 virus 3d p.i., there was pulmonary congestion, proliferation of bronchial epithelial cells in tertiary bronchus, cellular inflammatory infiltration in the lamina propria, and thickening of pulmonary capillary with red blood cell. B, infected by E1 virus 7d p.i., there was pulmonary congestion, thickening of small blood vessels and pulmonary capillary filled with red blood cell. C, infected by C12 virus 3d p.i., there was lung interlobular necrosis and inflammatory cells aggregation. D, infected by C12 virus 7d p.i., there was pulmonary slight gore, a small amount of pulmonary capillary serous fluid exudation.
(TIF)
