# Supplementary figures and images for: Genotype Diversity of H9N2 Viruses Isolated from Wild Birds and Chickens in Hunan Province, China
Source: PLoS One. 2014 Jun 30;9(6):e101287. doi: 10.1371/journal.pone.0101287 (PMC4076334; doi:10.1371/journal.pone.0101287)

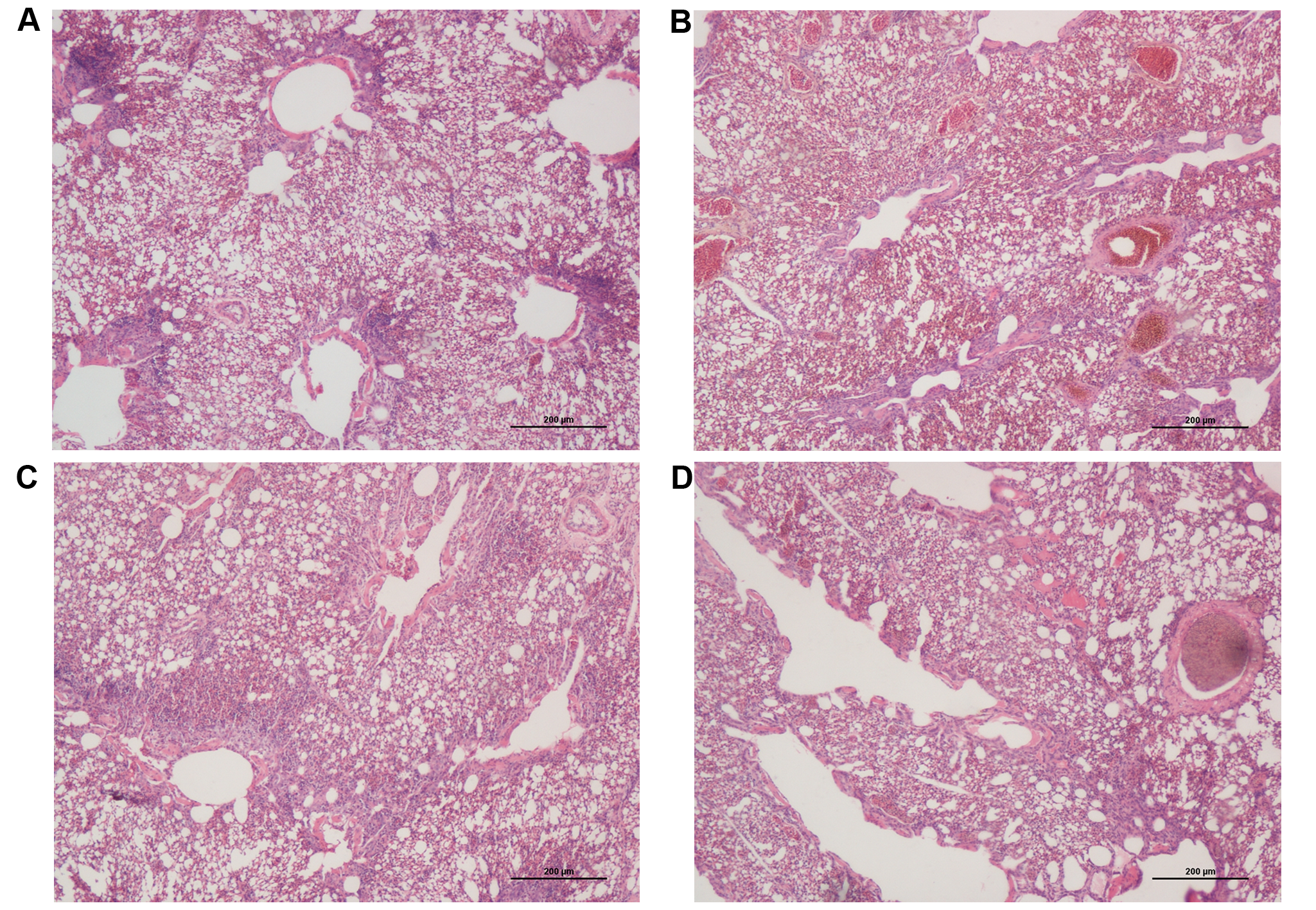

Supplement: Figure S1 — Histopathological changes of lungs are shown by Hematoxylin and Eosin (H&E) staining after virus infection. A, infected by E1 virus 3d p.i., there was pulmonary congestion, proliferation of bronchial epithelial cells in tertiary bronchus, cellular inflammatory infiltration in the lamina propria, and thickening of pulmonary capillary with red blood cell. B, infected by E1 virus 7d p.i., there was pulmonary congestion, thickening of small blood vessels and pulmonary capillary filled with red blood cell. C, infected by C12 virus 3d p.i., there was lung interlobular necrosis and inflammatory cells aggregation. D, infected by C12 virus 7d p.i., there was pulmonary slight gore, a small amount of pulmonary capillary serous fluid exudation. (TIF) [file pone.0101287.s001.tif]
